# Circadian Rhythm Traits Matter More Than Shift Work Demands in the Sleep–Depression–Safety Behavior Pathway Among Shift‐Working Nurses

**DOI:** 10.1155/jonm/9953116

**Published:** 2026-04-24

**Authors:** Huihan Zhao, Yu He, Qiuxia Li, Shuyun Wang, Lin Lin, Zhaoquan Huang

**Affiliations:** ^1^ School of Information and Management, Guangxi Medical University, No. 22 Shuangyong Road, 530021, Nanning, Guangxi, China, gxmu.edu.cn; ^2^ Department of Hematology, The First Affiliated Hospital of Guangxi Medical University, No. 6 Shuangyong Road, 530021, Nanning, Guangxi, China, gxmu.edu.cn; ^3^ University Engineering Research Center of Digital Medicine and Healthcare, Guangxi Medical University, No. 22 Shuangyong Road, 530021, Nanning, Guangxi, China, gxmu.edu.cn; ^4^ Key Laboratory of Hematology, Education Department of Guangxi Zhuang Autonomous Region, Guangxi Medical University, Nanning, Guangxi, China, gxmu.edu.cn; ^5^ Department of Clinical Laboratory, The First Affiliated Hospital of Guangxi Medical University, No. 6 Shuangyong Road, 530021, Nanning, Guangxi, China, gxmu.edu.cn; ^6^ Department of Health Management and Division of Physical Examination, The First Affiliated Hospital of Guangxi Medical University, No. 6 Shuangyong Road, 530021, Nanning, Guangxi, China, gxmu.edu.cn; ^7^ Department of Pathology, The First Affiliated Hospital of Guangxi Medical University, No. 6 Shuangyong Road, 530021, Nanning, Guangxi, China, gxmu.edu.cn; ^8^ The Academician Workstation on Metabolism and Health, The First Affiliated Hospital of Guangxi Medical University, No. 6, Shuangyong Road, Nanning, 530021, Guangxi, China, gxmu.edu.cn

**Keywords:** circadian rhythm, depression, safety behavior, shift work, sleep

## Abstract

**Objectives:**

The study examined how shift work demands and circadian rhythm traits influence the sleep–depression–safety behavior chain in shift‐working nurses.

**Methods:**

A cross‐sectional study was conducted from May 1, 2024, to May 31, 2025, recruiting shift‐working nurses from a tertiary hospital. Their circadian traits, sleep quality, depressive symptoms, and safety behavior were assessed using the Circadian Type Inventory (CTI, measuring flexible–rigid [FR] and languid–vigorous [LV]), the Morningness–Eveningness Questionnaire‐5 (MEQ‐5), the Pittsburgh Sleep Quality Index (PSQI), the Patient Health Questionnaire‐9 (PHQ‐9), and the Safety Behavior Questionnaire (SBQ). Objective shift work demands (total night shift count, shift work hours, shift workload exposure, and shift schedule entropy) were calculated based on data from the nursing management system. Piecewise structural equation modeling (SEM) and generalized additive models (GAMs) were used for data analysis.

**Results:**

The optimal SEM explained 22% of the variance in sleep quality, 44% in depressive symptoms, and 22% in safety behavior level. Languidity (higher LV score) was strongly associated with both poor sleep quality (*β* = 0.29) and depressive symptoms (*β* = 0.28), whereas flexibility (higher FR score) was positively associated with safety behavior, exerting both direct (*β* = 0.21) and indirect (*β* = 0.04) effects. Depressive symptoms showed the strongest negative association with safety behavior (*β* = −0.27). Circadian rhythm traits showed stronger associations with the sleep–psychological–safety behavior chain than shift work demands, which were mainly associated with safety behavior and only weakly with sleep quality and depressive symptoms. GAMs revealed that five key predictors (shift schedule entropy, FR, chronotype, sleep quality, and depressive symptoms) had predominantly linear effects on safety behavior.

**Conclusion:**

Circadian rhythm traits should be prioritized in shift scheduling to optimize the sleep–psychological–behavioral pathway, while balancing shift work demands may help improve their safety behavior among shift‐working nurses.

## 1. Introduction

Nurses constitute a vital component of the healthcare workforce and serve as the direct executors of clinical care. To ensure the continuity of healthcare provision, most nurses are required to work around‐the‐clock, with shift work being unavoidable. Shift work is commonly defined as working outside the standard hours of 7:00 a.m. to 6:00 p.m., although the definition varies across studies [1]. Shift models include fixed early, evening, or night shifts as well as regular or irregular rotations [2, 3]. Shift work often leads to irregular sleep–wake cycles and chronic sleep deprivation, resulting in circadian rhythm disruption. Circadian rhythms regulate a wide array of cellular, physiological, and behavioral processes, and their disruption further contributes to disease development and exacerbates existing conditions [4, 5]. Therefore, it is not surprising that shift work imposes profound adverse effects on nurses’ physical, psychological, and behavioral health.

The adverse impact of shift work on nurses is extensive and profound, impairing individual health and performance, and contributing to work errors, adverse events, and compromised patient safety [6–9]. In terms of health, sleep disturbance is the most prevalent issue [4, 10]. Shift workers often attempt to sleep during the day when the circadian wake‐promotion signal is at its peak, leading to difficulty initiating and maintaining sleep, resulting in fragmented sleep and accumulating homeostatic sleep debt [11]. Therefore, most shift workers report reduced subjective sleep quality and shorter sleep duration [4]. The prevalence of shift work sleep disorder among nurses varies widely across studies, ranging from 35.2% to 58.1% [12–14]. Increasing evidence also highlights that disrupted circadian rhythms and pronounced sleep disturbances frequently underlie the mental health consequences of shift work [15–17]. Besides, circadian rhythmicity plays a crucial role in the etiology and pathophysiology of depression [18]. Recent meta‐analyses and systematic reviews further demonstrate that shift work is associated with an increased overall risk of adverse mental health outcomes, particularly depressive symptoms [19–21]. The prevalence of depressive symptoms among nurses ranges from 10.6% to 58.8% across studies [14, 22, 23]. Accordingly, shift‐working nurses face chronic challenges to sleep and mental health, while the sleep–depression relationship remains complex and warrants further investigation.

At the same time, the chronic sleep and psychological problems often trigger a cascade of sleep–psychological–behavioral consequences that warrant urgent attention. Safety behavior is the safety‐related actions performed by individuals in an organization. Safety behavior has two dimensions: safety compliance and safety participation [24]. Evidence indicates that shift workers exhibit higher rates of workplace errors, likely driven by poor sleep quality and depressed mood, which impair cognitive efficiency and work performance [25]. Panagioti et al. further showed that mental health problems among healthcare workers, such as burnout and depression, nearly double the risk of patient safety incidents and unprofessional behavior [26]. Previous research has indicated that negative psychological states diminish motivation and responsiveness, undermining safety compliance and participation [27, 28]. Moreover, excessive workload depletes individual resources, damages physical and psychological health, and weakens safety motivation and behaviors [29]. For shift‐working nurses, prolonged exposure to heavy shift workloads further exhausts sleep and psychological resources, thereby reinforcing a cascade of sleep–psychological–behavioral problems. Uncovering the key influence factors and underlying pathways of this process is imperative for both nurse health promotion and patient safety risk management.

Individual differences in shift work tolerance remain insufficiently understood. Accumulating evidence highlights the critical role of shift work demands in shaping health outcomes. Empirical findings further demonstrate that excessive workload, extended working hours, and emotional job demands significantly contribute to sleep disturbance among night‐shift workers [30]. Evidence from a systematic review indicates that frequent night shifts and quick rotations significantly increase the risk of shift work disorder, fatigue, depression, and other negative health outcomes in nurses [31]. A large multicenter study in China identified workload‐related factors—such as busyness during night shifts, working more than 40 h per week, and poor sleep before or after night shifts—as major contributors to depression and anxiety among nurses [22]. Consensus statements from the Working Time Society [32] also emphasize that occupational schedules and demands play a pivotal role in shift work tolerance, yet further investigation is needed to clarify dose–response patterns of exposure (e.g., type, intensity, and duration) and the complex interplay with individual chronotype differences.

A key question remains: why do some nurses under identical shift work conditions maintain wakefulness, recover sleep, and adapt more effectively? Evidence suggests that these differences may be rooted in genetically influenced circadian typology, which moderates shift work tolerance and serves as an important predictor of adaptation capacity [6, 11, 33]. Circadian rhythm traits are typically characterized along three dimensions: phase (chronotype), amplitude, and stability [34]. With respect to chronotype (morningness–eveningness), “morning larks” or early chronotypes tend to be most alert and functional in the early morning, whereas “night owls” or late chronotypes perform better during the late afternoon and evening. Numerous studies [35–37] have demonstrated that chronotype is a robust predictor of sleep and mental health outcomes in shift‐working nurses.

Meanwhile, Folkard et al. [38] introduced three key factors underlying individual differences in circadian rhythm adaptation to assess adjustment to shift work: (i) rigidity versus flexibility of sleeping habits, (ii) ability to overcome drowsiness, and (iii) morningness–eveningness. The first two factors provide the conceptual basis for modern circadian‐type measures, including the flexible–rigid (FR) and languid–vigorous (LV) dimensions. Higher FR reflects greater adaptability to irregular schedules, whereas higher LV indicates increased vulnerability to fatigue after night shifts, thereby elevating the risk of shift work sleep disorder [39–41]. Both empirical evidence and expert consensus emphasize that occupational health policies, interventions, and scheduling strategies should account for circadian rhythm traits [32, 42, 43]. As relatively stable physiological and behavioral traits, circadian rhythm traits not only determine individual responses to shift work demands but may also interact with scheduling structures to shape the onset and progression of shift work–related adverse outcomes. Accordingly, it is imperative to clarify how shift work demands, circadian rhythm traits, and their interplay shape nurses’ sleep, mental health, and safety behaviors.

The Job Demands–Resources (JD‐R) theory is a classical work design framework with two core dimensions (job demands and job resources) explaining how aspects of the work environment affect employee well‐being and performance [44]. It proposes a health impairment process, where excessive job demands deplete physiological, psychological, and cognitive resources, thereby negatively impacting health and motivational processes. Since 2017, Bakker and Demerouti further incorporated personal resources and job crafting into the JD‐R model, emphasizing the dynamic interplay between job and personal resources and the cascading effects of job crafting on resource enhancement [45]. Moreover, personal resources exhibit a “gain spiral” effect, not only directly enhancing work resources but also mediating the impact of job demands and resources on health impairment and work engagement [46]. Personal resources can also buffer the negative effects of job demands on employee health [47], meaning that individuals with higher personal resources (e.g., circadian adaptability) cope more effectively with shift work demands. Therefore, the JD‐R theory provides an important theoretical framework for the present study to explore how shift work demands and individual circadian traits resources jointly influence sleep quality and subsequent psychological‐behavioral cascades among shift‐working nurses.

In summary, this study employed two complementary modeling strategies to examine the key factors, potential pathways, and the functional forms through which shift work demands and circadian rhythm traits influence the sleep–depression–safety behavior chain in shift‐working nurses. Piecewise structural equation modeling (SEM) was applied to address: (1) between shift work demands and circadian typology, which is more strongly linked to the chain? (2) what are the main factors and pathways associated with the chain? Subsequently, generalized additive models (GAMs) were used to verify (3) whether the effects of main factors on nurses’ safety behavior follow linear or nonlinear patterns? The study aimed to explore the potential influence mechanisms, inform continuous or threshold/inflection‐point monitoring, and provide guidance for shift scheduling as well as nurse health and nursing safety risk surveillance.

## 2. Methods

### 2.1. Study Design and Participants

This study used the same study sample as our previous publication [48] but includes additional variables, such as chronotype, safety behavior, and shift schedule entropy (SSE), and employs distinct analytical methods to address a different research question concerning the sleep–depression–safety behavior chain in shift‐working nurses. This cross‐sectional observational study was conducted at a tertiary general hospital in Guangxi, China, from May 1, 2024, to May 31, 2025. The study protocol was approved by the Ethics Committee of the First Affiliated Hospital of Guangxi Medical University (No.: 2024‐S711‐01). Shift‐working nurses were recruited through on‐site presentations and online advertisements. Eligible participants were those who: (1) were aged 20–45 years, (2) had been employed full time in clinical nursing for more than 6 months, and (3) had been assigned to a fixed ward and engaged in at least one shift per month during the past 6 months. Nurses who had major physical illnesses (e.g., cancer, cardiovascular or cerebrovascular disease, and diabetes), severe psychiatric disorders (e.g., schizophrenia and bipolar disorder), had undergone major surgery, experienced major family life events (e.g., bereavement or divorce), were pregnant or breastfeeding, had taken ≥ 3 consecutive days of medical leave during the past 6 months, or had unavailable shift work data were excluded. Written informed consent was obtained from all participants. Based on general recommendations for SEM, a sample size of at least 200 has previously been suggested [49]. Therefore, the sample size of 288 in this study was deemed sufficient. This study was reported in accordance with the STROBE guidelines.

### 2.2. Measures and Data Collection

All eligible participants were invited to complete a paper‐based self‐report questionnaire. The questionnaire collected general information (sex, age, ethnicity, marital status, number of children, education level, years of work/shift work experience, and professional title) as well as responses to the Circadian Type Inventory (CTI), the Morningness–Eveningness Questionnaire‐5 (MEQ‐5), the Pittsburgh Sleep Quality Index (PSQI), the Patient Health Questionnaire‐9 (PHQ‐9), and the Safety Behavior Questionnaire (SBQ). Questionnaires were distributed on site by the principal investigator and checked for completeness upon collection.

#### 2.2.1. Circadian Rhythm Stability and Amplitude

Circadian rhythm stability and amplitude were assessed using the CTI, an 11‐item instrument comprising two subscales: FR, 5 items, reflecting rhythm stability, and LV, 6 items, reflecting rhythm amplitude [50]. The Chinese version of the CTI, translated and validated by Qi et al. [34], has shown good psychometric properties, with Cronbach’s *α* of 0.80 for the FR and 0.73 for the LV subscale. Items are rated on a 5‐point Likert scale (1 = “almost never” to 5 = “almost always”). Higher FR scores indicate greater flexibility in adapting to irregular schedules, whereas higher LV scores reflect greater languidness and vulnerability to sleep loss. Individuals with scores ≥ 75th percentile on the FR and LV subscales were classified as flexible and languid types, respectively [50]. In the present study, internal consistencies were acceptable (Cronbach’s *α* = 0.848 for FR and 0.721 for LV subscale).

#### 2.2.2. Chronotype

Chronotype was assessed using the MEQ‐5, a simplified version of the original MEQ. The MEQ‐5 has demonstrated robust psychometric properties, including satisfactory construct validity, convergent validity, and test–retest reliability [51] and has been validated in Chinese populations with good reliability and validity [52, 53]. The scale consists of five items, yielding a total score ranging from 4 to 25, classifying individuals as evening type (4‐11 scores), intermediate type (12‐17 scores), or morning type (18‐25 scores). In the present study, the MEQ‐5 showed marginal internal consistency (Cronbach’s *α* = 0.627).

#### 2.2.3. Sleep Quality

Sleep quality over the past month was assessed using the Chinese version of the PSQI. The PSQI has demonstrated good internal consistency in previous studies (Cronbach’s *α* = 0.82–0.83) [54]. It consists of 19 items across seven components: subjective sleep quality, sleep latency, sleep duration, sleep efficiency, sleep disturbances, use of sleep medication, and daytime dysfunction. Each component is scored from 0 to 3, yielding a total score ranging from 0 to 21, with higher scores indicating poorer sleep quality. A PSQI total score > 7 has been shown to distinguish individuals with poor sleep quality, with high sensitivity (98.3%) and specificity (90.2%) [55]. In the present study, the PSQI demonstrated acceptable internal consistency (Cronbach’s *α* = 0.748). A total PSQI score > 7 was used as the threshold for poor sleep quality.

#### 2.2.4. Depressive Symptoms

Depressive symptoms over the past month were assessed using the PHQ‐9, a widely used self‐report instrument validated in Chinese populations by Zhang et al. [56] with high internal reliability (Cronbach’s *α* = 0.854). The PHQ‐9 consists of 9 items rated on a 4‐point scale (0 = “not at all” to 3 = “nearly every day”), yielding a total score ranging from 0 to 27, with higher scores indicating more severe depressive symptoms. A PHQ‐9 total score ≥ 10 is the internationally accepted cutoff for screening‐positive depressive symptoms, with both sensitivity and specificity of 0.85 [57]. In the present study, a cutoff of PHQ‐9 ≥ 10 was used to define screening‐positive depressive symptoms, and the scale demonstrated good internal consistency (Cronbach’s *α* = 0.871).

#### 2.2.5. Nurse Safety Behavior

Nurse safety behavior was measured with the SBQ, translated and validated in Chinese by Rong [58], which demonstrated high internal consistency (Cronbach’s *α* = 0.915). The scale is designed to measure healthcare workers’ behaviors aimed at preventing or minimizing patient harm. It is a unidimensional instrument consisting of 12 items rated on a 5‐point Likert scale (1 = “never” to 5 = “always”), with total scores ranging from 12 to 60. Higher scores indicate better safety behavior. In the present study, the scale also showed good reliability (Cronbach’s *α* = 0.845).

#### 2.2.6. Shift Work Demands

Shift work demands—including shift work quantity, shift workload intensity, and SSE—during the past 4 weeks were assessed. All data were extracted from the hospital nursing management system or computed as needed.

Shift work quantity was assessed by extracting the total night shift count (TNSC), total evening shift count (TESC), the combined total shift count (night + evening shifts, TSC), and total shift work hours (SWH). Shift periods were defined as follows: an evening shift was 18:00–00:00 with ≥ 4 consecutive working hours; a night shift was 00:00–08:00 with ≥ 4 consecutive working hours; and a long night shift was 18:00–08:00 with a 3‐h nap included, which was categorized as a night shift.

Shift workload intensity was assessed using workload (expected nurse‐to‐patient ratio (NPR)/actual NPR) [59, 60] and workload exposure (workload × working hours). The actual NPR for day and shift work during the observation period was calculated as the ratio of active primary nurses to assigned patients and extracted from the hospital’s nursing management system. The expected NPR for each ward was determined based on the disease severity classification proposed by Welton et al. [60]: mild severity (0), expected NPR = 1:8; moderate severity (1), expected NPR = 1:3; severe condition (2), expected NPR = 1:1. Given the exponential relationship between disease severity and expected NPR, the function was fitted as: expected NPR = 0.1154 × exp (1.0791 × severity score). Patient severity was represented by the Case Mix Index (CMI), a proxy for disease severity, clinical complexity, and resource consumption [61]. Unit‐level CMI data during the observation period were extracted from the Chinese CHS‐DRGs system and standardized to a 0–2 range to calculate expected NPR. Shift/day workload exposure = workload × shift/day work hours, with workload defined as the ratio of expected to actual NPRs for the corresponding shift or day work period during the observation period.

SSE was used to quantify the irregularity of shift distributions. Entropy‐based measures have been widely applied to quantify complexity in biological, social, and behavioral systems [62, 63]. Entropy measures, including those considered diversity indices, are fundamentally label‐independent, relying solely on the underlying probability distribution *p* rather than on the specific symbols of the labels [64]. Therefore, using an information‐theoretic approach derived from Shannon’s entropy, the proportion (*p*_*i*_) of each shift type (e.g., day, evening, night shift) within the observation window was calculated. The term log (*p*_*i*_) represents the information content of that shift type, and *p*_*i*_ log (*p*_*i*_) reflects its contribution of each shift type to the overall entropy. Shannon’s entropy [65] was then computed as follows:

(1) SSE=−∑i=1kpilogpi,

where *k* is the number of distinct shift types. To ensure comparability, the entropy value was normalized to the range [0, 1]. Higher values of SSE indicate more dispersed and irregular shift patterns, capturing variability beyond simple counts of different shifts.

### 2.3. Statistical Analysis

Data analysis was conducted using Python and R statistical packages. For descriptive statistics, normally distributed continuous variables were presented as mean ± standard deviation, skewed continuous variables as median and interquartile range (IQR), and categorical variables as frequencies and percentages.

#### 2.3.1. Univariate Analysis

The Mann–Whitney U test or Kruskal–Wallis H test was used to compare PSQI, PHQ‐9, and SBQ scores across groups defined by demographic characteristics, shift work demands, and circadian rhythm traits. Spearman’s rank correlation was employed to assess associations between PSQI, PHQ‐9, and SBQ scores and other continuous variables.

#### 2.3.2. Piecewise SEM

The piecewise SEM is particularly appropriate for capturing linear and nonlinear associations between predictors and response variables in empirical data [66]. In this study, piecewise SEM was applied to identify the key associated factors and potential pathways within the sleep–psychological–behavioral chain in shift‐working nurses. Response variables included PSQI, PHQ‐9, and SBQ scores. Predictors comprised observed indicators of shift work demands (TNSC, SWH, SWE, and SSE) as well as circadian rhythm traits (FR, LV, MEQ‐5 scores), relevant interaction terms, and covariates identified as significant in univariate analyses.

All continuous predictors were transformed using the Yeo–Johnson method and standardized (z‐scores) prior to modeling. The Yeo–Johnson transform [67] is a statistical method used to stabilize variance and make the data more normally distributed. It can be applied to a wider range of datasets, improving model performance by reducing the impact of skewed data. Multicollinearity assessment led to the exclusion of TSC due to high collinearity with TNSC and SWH. Given strong collinearity among age, work experience, and shift work experience, as well as its close association with professional title, age was retained as the representative variable.

Modeling was performed using the piecewise SEM package in R (v4.4.2). Model adequacy was assessed with Fisher’s C test, and iterative modifications were made based on pathway significance (*p* < 0.05), model fit (0 ≤ Fisher’s C/df ≤ 2 and 0.05 < *p* ≤ 1.00) and results of tests of directed separation [68]. Final path diagrams visualized significant directional relationships, with standardized path coefficients indicating the strength and direction of effects.

#### 2.3.3. GAMs

To evaluate whether nonlinear modeling improved explanatory performance in predicting SBQ score, we fitted GAMs to examine the linearity or nonlinearity of identified predictors and quantify their marginal contributions to SBQ score. All continuous variables were transformed using the Yeo–Johnson method and standardized. We used the pygam package in Python to fit fully linear, fully nonlinear, and partially nonlinear models. All spline terms were set to n_splines = 6 to allow adequate flexibility. In partially nonlinear models, each predictor was modeled with a spline, while the others remained linear. Model performance was evaluated using pseudo *R* ^2^, generalized cross‐validation (GCV) error, Akaike Information Criterion (AIC), and estimated degrees of freedom (EDoF). Predictors with EDoF ≈ 1 and minimal improvement in performance were considered linear. Partial effect plots were generated to visualize predictor‐outcome relationships. To quantify each variable’s marginal contribution, a stepwise inclusion strategy (SSE ⟶ FR ⟶ MEQ‐5 ⟶ PHQ‐9 ⟶ PSQI) was applied based on optimal linear or nonlinear specifications, with cumulative deviance explained calculated at each step.

## 3. Results

### 3.1. Descriptive Statistics

A total of 312 shift‐working nurses were recruited. After excluding 24 participants (10 pregnant, 4 lactating, 2 with diabetes, 3 postsurgical, 2 with sickness absence > 3 days, and 3 without shift data), 288 shift‐working nurses were included in the analysis, comprising 30 males (10.42%) and 258 females (89.58%). The median age was 33.5 years (range: 24–44). In the past 4 weeks, the median number of shifts was 5 (IQR:4), median SWH 47.5 (IQR:12), shift workload exposure 146.89 (IQR:132.10), and SSE 1.848 (IQR:0.399). The median FR score was 12.5 (IQR:6), with 26.4% classified as flexible type; the median LV score was 19 (IQR:6), with 26.0% classified as languid type. The median MEQ‐5 score was 13 (IQR:4), with 60.1% intermediate, 31.6% evening, and 8.3% morning types. Median PSQI and PHQ‐9 scores were 8 (IQR:4) and 7 (IQR:5), respectively, while the median SBQ score was 54.5 (IQR:7). Overall, 51.4% of nurses met criteria for poor sleep quality, 24.3% for screening‐positive depressive symptoms, and 20.1% for both conditions. Detailed descriptive statistics are presented in Supporting Table S1. Common method bias analysis showed that the first factor accounted for 17.7% of the variance, well below the 40% threshold, indicating no significant common method bias in this study.

### 3.2. Univariate Analysis

(1) Basic characteristics: Sleep quality and depressive symptoms showed no significant difference across most demographic subgroups. However, sleep quality showed marginal differences across age groups (*p* = 0.085), and depressive symptoms showed a marginal difference across marital status groups (*p* = 0.061). In contrast, safety behavior levels differed significantly by gender (*p* = 0.008), age (*p* = 0.011), marital status (*p* = 0.036), work experience (*p* = 0.024), shift work experience (*p* = 0.045), and professional title (*p* = 0.037) (Table 1). (2) Shift work and circadian rhythm characteristics: sleep quality differed significantly by total shift count (*p* = 0.032), LV type (*p* < 0.001), and chronotype (*p* < 0.001). Depressive symptoms differed significantly by FR type (*p* = 0.016), LV type (*p* < 0.001), and chronotype (*p* = 0.041). Safety behavior levels differed significantly by FR type (*p* = 0.029) (Table 2). (3) Correlation analysis: BMI, depressive symptoms, LV, and MEQ‐5 scores were significantly correlated with sleep quality (*p* < 0.05). Safety behavior levels, FR, LV, and MEQ‐5 scores were significantly correlated with depressive symptoms (*p* < 0.05). Age, years of experience, shift work experience, FR, and MEQ‐5 scores were significantly correlated with safety behavior levels (*p* < 0.05) (details in Table 3).

**TABLE 1:** Sociodemographic characteristics and group differences in PSQI, PHQ‐9, and SBQ score.

Variables	*N* (%)	PSQI	*p* value	PHQ‐9	*p* value	SBQ	*p* value
		Median (IQR)		Median (IQR)		Median (IQR)	
Gender							
Male	30 (10.4)	6.0 (3.75)	0.162	8.0 (5.75)	0.957	50.5 (7.75)	**0.008**
Female	258 (89.6)	8.0 (4.0)		7.0 (5.0)		55.0 (6.0)	
Age range							
20 ≤ years < 30	90 (31.3)	7.5 (4.0)	0.085	7.0 (4.0)	0.536	53.0 (7.0)	**0.011**
30 ≤ years < 40	167 (58.0)	8.0 (4.0)		7.0 (5.0)		55.0 (5.5)	
years ≥ 40	31 (10.7)	6.0 (3.5)		6.0 (6.5)		56.0 (5.0)	
BMI							
BMI < 18.5	18 (6.3)	8.5 (3.5)	0.175	7.0 (2.75)	0.750	53.5 (6.75)	0.078
18.5 ≤ BMI < 24	218 (75.7)	8.0 (4.0)		7.0 (5.0)		55.0 (6.0)	
24 ≤ BMI < 28	48 (16.7)	7.0 (4.0)		7.0 (5.25)		54.0 (9.0)	
BMI ≥ 28	4 (1.4)	7.5 (4.5)		10.0 (4.75)		47.0 (6.5)	
Ethnicity							
Han	170 (59.0)	8.0 (4.0)	0.787	7.0 (5.75)	0.883	54.0 (6.0)	0.100
Zhuang minority	104 (36.1)	8.0 (4.0)		7.0 (5.0)		56.0 (6.25)	
Other	14 (4.9)	7.5 (4.0)		7.0 (5.5)		53.0 (9.5)	
Marital status							
Married	186 (64.6)	8.0 (4.0)	0.525	7.0 (5.0)	0.061	55.0 (5.0)	**0.036**
Unmarried	96 (33.3)	8.0 (4.0)		7.0 (4.0)		53.0 (7.0)	
Divorced	6 (2.1)	9.0 (2.75)		10.0 (0.75)		53.0 (3.5)	
Education level							
Associate degree	9 (3.1)	9.0 (2.0)	0.983	6.0 (4.0)	0.809	52.0 (10.0)	0.258
Bachelor’s degree	262 (91.0)	8.0 (4.0)		7.0 (5.0)		55.0 (6.75)	
Master’s degree or higher	17 (5.9)	7.0 (4.0)		6.0 (5.0)		51.0 (6.0)	
Work experience							
years ≤ 5	71 (24.7)	7.0 (4.0)	0.444	7.0 (4.5)	0.938	53.0 (7.0)	**0.024**
5 < years ≤ 10	69 (24.0)	7.0 (4.0)		7.0 (5.0)		55.0 (8.0)	
10 < years ≤ 15	93 (32.3)	9.0 (4.0)		7.0 (4.0)		55.0 (5.0)	
15 < years ≤ 20	46 (16.0)	7.0 (3.75)		6.0 (7.0)		56.0 (6.0)	
Years > 20	9 (3.1)	6.0 (4.0)		8.0 (4.0)		57.0 (3.0)	
Professional title							
Junior and below	97 (33.7)	7.0 (4.0)	0.684	7.0 (4.0)	0.708	53.0 (7.0)	**0.037**
Intermediate grade	187 (64.9)	8.0 (4.0)		7.0 (6.0)		55.0 (5.5)	
Senior	4 (1.4)	9.0 (5.0)		6.0 (1.5)		56.5 (1.5)	
Number of children							
0	117 (40.6)	8.0 (4.0)	0.617	7.0 (5.0)	0.104	54.0 (8.0)	0.349
1	73 (25.3)	8.0 (4.0)		8.0 (5.0)		55.0 (7.0)	
2	96 (33.3)	7.0 (4.25)		6.0 (5.0)		55.0 (5.0)	
3	2 (0.7)	6.0 (3.0)		9.0 (3.0)		56.0 (3.0)	
Shift work experience							
years ≤ 5	80 (27.8)	7.0 (4.0)	0.452	7.0 (4.25)	0.921	53.0 (7.0)	**0.045**
5 < years ≤ 10	86 (29.9)	8.0 (4.0)		7.0 (5.0)		55.0 (7.0)	
10 < years ≤ 15	76 (26.4)	8.0 (4.0)		7.0 (4.0)		55.0 (5.0)	
15 < years ≤ 20	40 (13.9)	7.0 (4.0)		6.0 (7.25)		56.0 (5.25)	
Years > 20	6 (2.1)	7.5 (3.25)		8.0 (3.75)		56.0 (3.5)	
Night‐shift off days							
0.5 ≤ Off days < 2	7 (2.4)	8.0 (2.5)	0.052	8.0 (4.5)	0.628	58.0 (11.5)	0.784
2 ≤ Off days < 3	228 (79.2)	8.0 (4.0)		7.0 (5.0)		54.0 (7.0)	
Off days ≥ 3	53 (18.4)	7.0 (4.0)		6.0 (5.0)		55.0 (4.0)	

*Note:* PSQI, Pittsburgh Sleep Quality Index (sleep quality); PHQ‐9, Patient Health Questionnaire‐9 (depressive symptoms). Bold values indicate *p* values with statistical significance (*p* < 0.05).

Abbreviations: BMI, body mass index; IQR, interquartile range; SBQ, Safety Behavior Questionnaire.

**TABLE 2:** Comparisons of shift work and circadian rhythm characteristics by PSQI, PHQ‐9, and SBQ scores.

Variables	*N* (%)	PSQI	*p* value	PHQ‐9	*p*value	SBQ	*p* value
		Median (IQR)		Median (IQR)		Median (IQR)	
Shift work characteristics							
Shift model							
DN	188 (65.3)	8.0 (5.0)	0.951	7 (6.0)	0.782	55.0 (5.0)	0.087
APN	100 (34.7)	8.0 (4.0)		7 (5.0)		53.5 (8.0)	
Total shift count							
Shift count ≤ 2	13 (41.5)	8.0 (8.0)	**0.032**	7.0 (6.0)	0.562	55.0 (6.0)	0.689
2 < Shift count ≤ 4	105 (36.5)	7.0 (4.0)		6.0 (5.0)		55.0 (5.0)	
4 < Shift count ≤ 8	124 (43.1)	8.0 (4.0)		7.0 (6.0)		55.0 (7.0)	
Shift count > 8	46 (16.0)	8.0 (4.0)		8.0 (4.0)		53.0 (9.0)	
Total night shift count							
Night shift count ≤ 2	28 (9.7)	8.0 (6.0)	0.078	7.5 (5.0)	0.435	55.0 (7.5)	0.736
2 < night shift count ≤ 4	190 (66.0)	7.0 (5.0)		7.0 (5.0)		54.0 (7.0)	
Night shift count > 4	70 (24.3)	9.0 (5.0)		8.0 (5.75)		54.0 (8.0)	
Shift work hours							
Shift work hours ≤ 22	14 (4.9)	7.5 (8.25)	0.085	5.5 (5.5)	0.479	53.5 (6.75)	0.386
22 < Shift work hours ≤ 44	123 (42.7)	7.0 (5.0)		7.0 (5.0)		55.0 (5.0)	
Shift work hours > 44	151 (52.4)	8.0 (4.0)		7.0 (5.5)		54.0 (8.0)	
Shift workload exposure							
Shift workload exposure ≤ 67.1	70 (25.2)	8.0 (4.0)	0.404	6.5 (4.0)	0.456	54.5 (8.0)	0.434
67.1 < Shift workload exposure ≤ 146.9	69 (24.8)	7.0 (4.0)		8.0 (4.0)		55.0 (6.0)	
146.9 < Shift workload exposure ≤ 199.2	69 (24.8)	7.0 (4.0)		7.0 (5.0)		54.0 (7.0)	
Shift workload exposure > 199.2	70 (25.2)	9.0 (5.0)		7.0 (6.0)		55.0 (5.0)	
Circadian rhythm characteristics							
FR type							
Rigid	212 (73.6)	8.0 (4.0)	0.642	7.0 (6.0)	**0.016**	54.0 (7.0)	**0.029**
Flexible	76 (26.4)	7.0 (4.25)		6.0 (4.0)		55.5 (5.25)	
LV type							
Vigorous	213 (74.0)	7.0 (4)	**< 0.001**	6.0 (4.0)	**< 0.001**	55.0 (7.0)	0.454
Languid	75 (26.0)	10.0 (5)		9.0 (5.0)		54.0 (7.0)	
Chronotype							
Intermediate	173 (60.1)	8.0 (4.0)	**< 0.001**	7.0 (5.0)	**0.041**	55.0 (6.0)	0.229
Evening‐type	91 (31.6)	9.0 (5.0)		7.0 (4.5)		54.0 (8.0)	
Morning‐type	24 (8.3)	5.0 (3.25)		4.5 (4.25)		54.5 (5.0)	

*Note:* PSQI, Pittsburgh Sleep Quality Index (sleep quality); PHQ‐9, Patient Health Questionnaire‐9 (depressive symptoms); DN, day‐night shift; APN, day (AM)‐evening (PM)‐night (N) shift. Shift‐work stratification was based on the DN model, where nurses typically worked one 11‐h night shift per week. Thus, four night‐shifts and 44 working hours over four weeks were set as reference thresholds, with higher and lower categories defined accordingly. Workload exposure was divided into quartiles. Bold values indicate *p* values with statistical significance (*p* < 0.05).

Abbreviations: BMI, body mass index; IQR, interquartile range; SBQ, Safety Behavior Questionnaire.

**TABLE 3:** Spearman’s correlations of key continuous variables in the study.

Variables	PSQI score		PHQ‐9 score		SBQ score	
	*p* value	Spearman’s *ρ*	*p* value	Spearman’s *ρ*	*p* value	Spearman’s *ρ*
Total night shift count	0.523	0.038	0.559	0.035	0.586	−0.032
Total evening shift count	0.129	0.090	0.410	0.049	0.063	−0.110
Total shift count	0.080	0.103	0.272	0.065	0.124	−0.091
Shift work hours	0.074	0.106	0.215	0.073	0.118	−0.092
Day work hours	0.749	0.019	0.962	0.003	0.134	0.088
Day workload	0.458	0.045	0.457	−0.045	0.112	0.095
Shift workload	0.095	0.100	0.859	0.011	0.194	0.078
Day workload exposure	0.513	0.039	0.911	−0.007	0.081	0.105
Shift workload exposure	0.083	0.104	0.684	0.024	0.352	0.056
Age	0.342	−0.056	0.420	−0.048	**0.015**	**0.144**
Years of experience	0.404	−0.049	0.510	−0.039	**0.002**	**0.178**
Shift work experience	0.377	−0.052	0.621	−0.029	**0.004**	**0.171**
BMI	**0.036**	**−0.123**	0.640	0.028	0.374	−0.053
Shift schedule entropy	0.065	0.109	0.152	0.085	0.051	−0.115
PSQI score	/	/	**0.000**	**0.559**	0.869	0.010
PHQ‐9 score	**0.000**	**0.559**	/	/	**0.000**	**−0.224**
FR score	0.873	−0.009	**0.002**	**−0.179**	**0.000**	**0.205**
LV score	**0.000**	**0.358**	**0.000**	**0.413**	0.196	−0.076
MEQ‐5 score	**0.000**	**−0.272**	**0.012**	**−0.149**	**0.020**	**0.137**
SBQ score	0.869	0.010	**0.000**	**−0.224**	/	/

*Note: ρ* = Spearman’s rank correlation coefficient; *p* values are shown for each test. PSQI, Pittsburgh Sleep Quality Index (sleep quality); PHQ‐9, Patient Health Questionnaire‐9 (depressive symptoms). Bold values indicate correlation coefficients and *p* values that are statistically significant (*p* < 0.05).

Abbreviations: BMI, body mass index; FR, flexible–rigid; LV, languid–vigorous; MEQ‐5, Morningness–Eveningness Questionnaire‐5; SBQ, Safety Behavior Questionnaire.

### 3.3. SEMs

**FIGURE 1:**
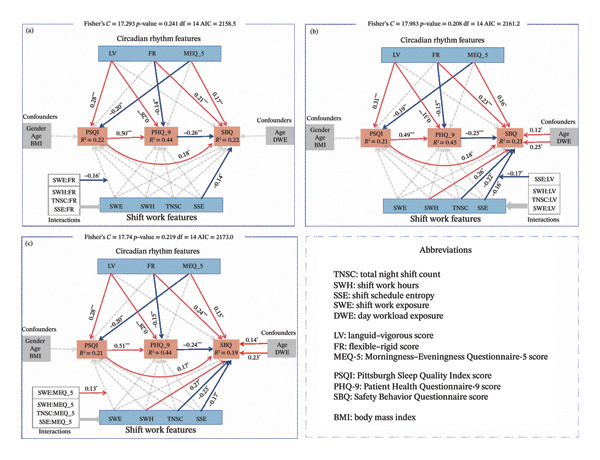
Path models of shift work and circadian rhythm type on sleep, depression, and safety behavior. The figure presents the three final piecewise SEM models. Model 1 (a) pSEM with FR as the moderator; Model 2 (b) pSEM with LV as the moderator; Model 3 (c) pSEM with MEQ‐5 as the moderator. Predictors were grouped into shift‐work features (SWE, SSE, SWH, and TNSC) and circadian rhythm features (FR, LV, MEQ‐5), with gender, age, BMI, and DWE included as covariates. All paths are displayed in the models; however, only significant paths are annotated with standardized coefficients (^∗^ *p* < 0.05, ^∗∗^ *p* < 0.01, ^∗∗^ *p* < 0.001). Red arrows indicate positive associations, while blue arrows indicate negative associations. Boxes show *R* ^2^ values. Overall, compared with shift‐work demands, the models indicate that circadian rhythm traits are the predominant drivers of the sleep–depression–safety behavior chain among shift nurses.

Three piecewise SEMs were fitted, with circadian rhythm traits (FR, LV, and MEQ‐5) entered separately as moderators of the relationships between shift‐work demands and outcomes (Figure 1). In the optimal SEM (Figure 1(a)) with FR as the moderator, the model explained 22% of the variance in sleep quality, 44% in depressive symptoms, and 22% in safety behavior level. The key influence factor of poor sleep quality (higher PSQI score) was greater languidness (*β* = 0.29). Depressive symptoms were mainly influenced by poor sleep quality (*β* = 0.50) and greater languidness (*β* = 0.28). Safety behavior level was mainly associated with lower depressive symptoms (*β* = −0.27) and greater flexibility (higher FR score, *β* = 0.21). The main pathway influencing depressive symptoms was greater languidness ⟶ depressive symptoms (total effect = 0.42), consisting of a direct effect of 0.28 and an indirect effect of 0.14. The critical pathways influencing safety behavior were a negative direct effect from depressive symptoms to safety behavior level (total effect = −0.26) and a positive effect from greater flexibility to safety behavior level (total effect = 0.25), consisting of a direct effect of 0.21 and an indirect effect of 0.04. These identified significant factors and pathways remained relatively stable across the other two SEM models. Notably, shift‐work demands showed only direct effects on safety‐behavior level but no significant effects on sleep quality and depressive symptoms. A summary of the path coefficients is provided in Table 4. For clarity, only significant standardized path coefficients are displayed in Figure 1, whereas the complete SEM results (including nonsignificant paths across the three models) are presented in Table S2. Based on the optimal SEM (with FR as the moderator), the number of significant paths was 6 for circadian rhythm traits, compared to 2 for shift work demands, and the sum of absolute path coefficients was 1.28 for circadian rhythm traits versus 0.32 for shift work demands on the sleep–psychological–safety behavior chain. These findings indicate that circadian rhythm traits exert a stronger influence on the sleep–psychological–safety behavior chain than shift work demands.

**TABLE 4:** Pathways linking shift‐work and circadian rhythm characteristics to safety behavior (piecewise SEM models).

Paths	Model 1	Model 2	Model 3
Total effect: MEQ_5 ⟶ SBQ	**0.16**	**0.15**	**0.14**
Direct effect: MEQ_5 ⟶ SBQ	**0.17**	**0.16**	**0.15**
Indirect effect: MEQ_5 ⟶ PSQI ⟶ SBQ	**−0.04**	**−0.03**	**−0.03**
MEQ_5 ⟶ PSQI	−0.20	−0.19	−0.20
PSQI ⟶ SBQ	0.18	0.18	0.17
Indirect effect: MEQ_5 ⟶ PSQI ⟶ PHQ‐9 ⟶ SBQ	**0.03**	**0.02**	**0.02**
MEQ_5 ⟶ PSQI	−0.20	−0.19	−0.20
PSQI ⟶ PHQ_9	0.50	0.49	0.51
PHQ_9 ⟶ SBQ	−0.26	−0.25	−0.24
Total effect: FR ⟶ SBQ	**0.25**	**0.27**	**0.28**
Direct effect: FR ⟶ SBQ	**0.21**	**0.23**	**0.24**
Indirect effect: FR ⟶ PHQ‐9 ⟶ SBQ	**0.04**	**0.04**	**0.04**
FR ⟶ PHQ‐9	−0.14	−0.15	−0.15
PHQ‐9 ⟶ SBQ	−0.26	−0.25	−0.24
Total effect: LV ⟶ SBQ	**−0.06**	**−0.06**	**−0.05**
Indirect effect: LV ⟶ PHQ‐9 ⟶ SBQ	**−0.07**	**−0.08**	**−0.07**
LV ⟶ PHQ‐9	0.28	0.31	0.28
PHQ‐9 ⟶ SBQ	−0.26	−0.25	−0.24
Indirect effect: LV ⟶ PSQI ⟶ PHQ‐9 ⟶ SBQ	**−0.04**	**−0.04**	**−0.03**
LV ⟶ PSQI	0.28	0.31	0.28
PSQI ⟶ PHQ‐9	0.50	0.49	0.51
PHQ‐9 ⟶ SBQ	−0.26	−0.25	−0.24
Indirect effect: LV ⟶ PSQI ⟶ SBQ	**0.05**	**0.06**	**0.05**
LV ⟶ PSQI	0.28	0.31	0.28
PSQI ⟶ SBQ	0.18	0.18	0.17
Total effect: PSQI ⟶ SBQ	**0.05**	**0.06**	**0.05**
Direct effect: PSQI ⟶ SBQ	**0.18**	**0.18**	**0.17**
Indirect effect: PSQI ⟶ PHQ‐9 ⟶ SBQ	**−0.13**	**−0.12**	**−0.12**
PSQI ⟶ PHQ‐9	0.50	0.49	0.51
PHQ‐9 ⟶ SBQ	−0.26	−0.25	−0.24
Total effect: PHQ‐9 ⟶ SBQ (direct)	**−0.26**	**−0.25**	**−0.24**
Total effect: SSE ⟶ SBQ (direct)	**−0.14**	**−0.16**	**−0.17**
Total effect: TNSC ⟶ SBQ (direct)	/	**−0.22**	**−0.23**
Total effect: SWH ⟶ SBQ (direct)	/	**0.26**	**0.27**
Total effect: DWE ⟶ SBQ (direct)	/	**0.25**	**0.23**
Total effect: Age ⟶ SBQ (direct)	/	**0.12**	**0.14**

*Note:* Model 1: piecewise SEM with FR as the moderator; Model 2: piecewise SEM with LV as the moderator; Model 3: piecewise SEM with MEQ‐5 as the moderator. PSQI, Pittsburgh Sleep Quality Index (sleep quality); PHQ‐9, Patient Health Questionnaire‐9 (depressive symptoms); SBQ, Safety Behavior Questionnaire score; FR, flexible–rigid score; LV, languid–vigorous score; MEQ‐5, Morningness–Eveningness Questionnaire‐5 score. Bold values indicate the estimated coefficients of total, direct, and indirect effects for the corresponding pathways.

Abbreviations: BMI, body mass index; DWE, day workload exposure; SSE, shift schedule entropy; SWE, shift workload exposure; SWH, shift work hours; TNSC, total night shift count.

### 3.4. GAMs

**FIGURE 2:**
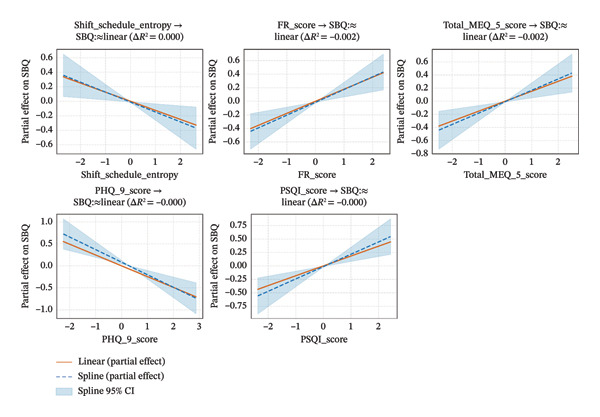
Partial effect plots comparing linear and nonlinear associations between five key predictors and SBQ scores. Each panel displays the partial effect of one predictor on SBQ, comparing linear fits (solid orange) and spline‐based fits (dashed blue) with 95% confidence intervals (shaded area). The change in pseudo *R* ^2^ (Δ*R* ^2^) was calculated as the difference in model fit when replacing the linear term of a predictor with a spline term while holding other predictors constant. All predictors showed Δ*R* ^2^ values below the predefined threshold of 0.005, indicating that their associations with SBQ were approximately linear. FR, MEQ, and PSQI scores were positively associated with SBQ, whereas PHQ‐9 scores and shift schedule entropy were negatively associated with SBQ score. SBQ, Safety Behavior Questionnaire score; PSQI, Pittsburgh Sleep Quality Index (sleep quality); PHQ‐9, Patient Health Questionnaire‐9 (depressive symptoms); FR, flexible–rigid score; MEQ‐5, Morningness–Eveningness Questionnaire‐5 score.

GAMs were fitted using both fully linear and fully spline‐based specifications for the five key predictors (SSE, FR, chronotype, depressive symptoms, and sleep quality) for SBQ identified in the optimal SEM. The full‐spline GAM yielded a slightly higher pseudo *R* ^2^ (0.1444) compared with the fully linear GAM (0.1423), with comparable GCV scores (0.908 vs. 0.906) and AIC (788.3 vs. 788.1), indicating limited gain in explanatory power from global nonlinear modeling. Variable‐specific nonlinearity was further tested, but improvements in model fit were negligible (Figure 2), suggesting that all five predictors were approximately linear. Consequently, a stepwise linear GAM was used to evaluate the cumulative explanatory contributions of the five key predictors and revealed a total pseudo *R* ^2^ of 14.23%, with FR accounting for the largest proportion of explained variance (4.74%), followed by sleep quality (3.42%), chronotype (2.30%), depressive symptoms (2.17%), and SSE (1.60%).

## 4. Discussion

This study employed two complementary modeling strategies‐piecewise SEM and GAM‐to ensure both explanatory clarity and modeling flexibility. The findings indicated that circadian rhythm traits were more strongly associated than shift work demands with the sleep–psychological–safety behavior chain in shift‐working nurses. Shift work demands had limited influence on sleep quality and depressive symptoms, exerting only direct effects on safety behavior. Greater flexibility and lower depressive symptoms were the primary factors associated with nurses’ safety behavior, with predominantly linear relationships. Taken together, these findings provide empirical support for prioritizing circadian traits in occupational health strategies, while also underscoring that integrating circadian traits with shift work demands may yield greater benefits for patient safety risk management.

### 4.1. Circadian Rhythm Traits Show Stronger Associations Than Shift Work Demands With the Sleep–Psychological–Safety Behavior Chain Among Shift‐Working Nurses

Although extensive evidence has demonstrated that both shift work demands and circadian rhythm traits influence nurses’ tolerance to shift work [32, 69], few studies have directly compared their relative effects. This has left clinical managers puzzled about which factors should be prioritized in shift scheduling. Recent evidence indicated that nurses with lower flexibility, higher languidness, and evening‐oriented chronotypes exhibit poorer tolerance to shift work [70]. The Working Time Society consensus statements also emphasize that late chronotype, low scores of languidness, and flexibility are consistently associated with perceived or actual shift work tolerance, although certain features of shift work, such as shift types, higher intensity, and longer duration, are also linked to poorer health outcomes [32]. Their causal pathways remain unclear. By integrating objective measures of shift work demands (quantity, intensity, and variability) with circadian rhythm traits (phase, stability, and amplitude), the present study provides novel evidence for clinical shift scheduling, suggesting that circadian rhythm traits should be prioritized over shift work demands in improving the sleep–psychological–safety behavior chain among shift‐working nurses.

Notably, shift work demands exerted limited influence on sleep quality and depression while significantly and directly affecting safety behavior. This contrasts with previous evidence linking nonday shifts, night shift density, and quick returns to higher risks of shift work sleep disorder [71, 72]. Similarly, a 4.5‐year prospective cohort of nurses further identified a dose–response relationship between night shift exposure and physician‐diagnosed sleep disorders, recommending that night shifts should not exceed 50 within a 6‐month period [73]. In addition, SEM has shown that workload strongly predicts job stress (*β* = 0.747) and negatively affects mental health (*β* = −0.291), physical health (*β* = −0.253), and sleep quality (*β* = −0.234) among nurses [74]. Moreover, depression and anxiety are highly prevalent among shift‐working nurses and are aggravated by heavy night shift workload, weekly working hours exceeding 40, and poor sleep quality before/after night shifts [22]. Although our findings appear inconsistent with these results, population heterogeneity may partly account for the discrepancy. More large‐scale studies are warranted to validate these associations.

At the same time, the observed negative associations of TNSC and SSE with safety behavior align with evidence from South Korea, where high job demands were found to undermine safety behavior [27], and with findings that nurses who had worked more than 10 weeks of night shifts in the previous year reported poorer performance and greater patient safety concerns [75]. Shift work is well known to induce circadian misalignment, impair cognitive functioning and alertness, and increase attentional errors. A study from France further demonstrated that ICU physicians experienced significant declines in multiple cognitive domains—including working memory, processing speed, perceptual reasoning, and cognitive flexibility—after a single night shift [76]. Moreover, shift work–related cognitive impairments can worsen across consecutive night shifts, depending on working conditions and the degree of circadian adaptation [77]. Building on this evidence, we speculate that a higher TNSC and greater SSE may aggravate circadian disruption and hinder adaptation, thereby intensifying misalignment and cognitive impairment and directly undermining safety behavior. However, dose–response models of shift work demand on nurses’ health and behavior still require further elucidation to inform job crafting and guide safety behavior promotion and patient safety risk management.

### 4.2. LV as a Key Factor Associated With Poor Sleep Quality and Depression Among Shift‐Working Nurses

It is well established that sleep disturbances can elicit or exacerbate symptoms in individuals at risk for mental health disorders. Prior reviews have emphasized that pronounced sleep disturbances frequently underlie the mental health consequences of shift work [15], and circadian disruption has been associated with various mood disorders, playing a role in the etiology and pathophysiology of depression [18]. Data from the NHANES further demonstrate a dose–response relationship between sleep patterns and the risk of clinically relevant depression [78]. Consistent with these findings, our results support the view that poor sleep quality is a primary influence factor of depressive symptoms among shift‐working nurses.

Beyond sleep, our analyses highlight that languidness (higher LV scores) emerges as a shared key factor of both poor sleep quality and depressive symptoms. Previous studies have consistently linked LV tendencies to the onset of shift work sleep disorder among shift workers [39, 41]. A prospective study of Chinese intern nurses identified morningness (protective), languidness (risk‐enhancing), and flexibility (protective) as significant predictors of both the onset and persistence of shift work sleep disorder [12]. Moreover, nurses classified as languid reported higher levels of burnout and job dissatisfaction compared to vigorous nurses [40]. Taken together, these findings suggest that greater languidness constitutes a trait‐level vulnerability that predisposes shift‐working nurses to shift work sleep disorder and broader mental health problems. Therefore, recognizing and monitoring LV tendency in clinical practice may help identify high‐risk nurses and inform tailored interventions or scheduling strategies to mitigate its adverse effects on sleep and mental health.

### 4.3. Depressive Symptoms and FR as Key Factors and Potential Pathways Related to Nurses’ Safety Behavior

In our study, depressive symptoms and FR emerged as the main factors associated with nurses’ safety behavior. The dominant pathways included a direct detrimental effect of depressive symptoms and a direct protective effect of FR on safety behavior. Moreover, the key predictors identified in our models (FR, chronotype, depressive symptoms, sleep quality, and SSE) predominantly influenced safety behavior through linear relationships.

This is in line with prior evidence that mental health has been identified as a predictor of safety behavior and depressed mood in shift workers was significantly related to increased workplace errors [25, 79]. This is not surprising: according to JD‐R theory, when shift work demands remain chronically high and resources are insufficient, employees may experience strain, negative emotions, and maladaptive self‐regulation, resorting to destructive strategies (e.g., avoidance coping and self‐undermining) that further compromise safety‐related behaviors [45]. Among shift‐working nurses, depressive symptoms may undermine communication, self‐regulation, and coping, thereby reducing compliance and participation in safety‐related behaviors. By contrast, empirical evidence directly linking flexibility with safety behavior remains limited. We speculate that its protective role may lie in greater adaptability to irregular schedules, which—either directly or through enhanced shift work tolerance—exerts a positive influence on safety behavior. This hypothesis requires further investigation. Taken together, these results suggest the importance of addressing depressive symptoms and fostering flexibility in shift‐working nurses as critical strategies for patient safety risk management.

Regarding the effect of sleep quality on nurses’ safety behavior, our findings diverge from previous studies. A study reported that poor sleep quality in shift workers was significantly associated with increased workplace errors [25]. Research focusing on construction workers also found a significant negative effect of PSQI scores on safety behavior (*β* = −0.189) [79], and data from the UK Biobank cohort demonstrated that short sleep duration predicted poorer performance in middle‐ and older‐aged adults (*β* = −0.24) [80]. A theoretical model likewise suggests that the relationship between insomnia and injury risk is mediated by its detrimental influence on safety behaviors [81]. By contrast, our study revealed a positive association between worse sleep quality and safety behavior. This counterintuitive finding may reflect compensatory mechanisms, whereby nurses experiencing poor sleep become more vigilant and cautious to avoid mistakes in a high‐pressure healthcare system, or it may be attributable to contextual factors specific to our sample. However, this explanation remains speculative and warrants further investigation.

## 5. Conclusions

Taken together, this study clarified that circadian rhythm traits exerted a stronger influence than shift work demands on the sleep–psychological–safety behavior chain among shift‐working nurses. LV as a key factor associated with poor sleep quality and depressive symptoms, whereas FR acted as a key protective factor influencing safety behavior. Depressive symptoms were identified as the dominant negative pathway undermining safety behavior. In contrast, shift work demands exerted only direct effects on safety behavior, with limited impact on sleep or depression. The effects of five key predictors (FR, chronotype, depressive symptoms, sleep quality, and SSE) had predominantly linear effects on safety behavior. These findings provide novel empirical evidence supporting the prioritization of circadian rhythm traits in shift scheduling to optimize the sleep–psychological–behavioral chain among nurses. Monitoring and mitigating depressive symptoms, fostering flexibility, and balancing shift work demands are critical strategies to enhance nurses’ safety behavior and safeguard patient safety.

## 6. Limitations

This study has several limitations that should be acknowledged. First, it employed a cross‐sectional, single‐center design, which limits causal inference. Future multicenter prospective cohort studies with larger samples are needed to validate the findings and further clarify causality. Second, circadian rhythm traits, sleep quality, depressive symptoms, and safety behavior were assessed using self‐reported questionnaires, which may introduce recall or reporting bias; future studies should incorporate objective measures (e.g., actigraphy, electronic scheduling logs, and wearable devices) to improve accuracy. Third, shift work demand variables were observed over only a 4‐week window in the present study, without capturing longer‐term dynamic changes. Future research with longitudinal monitoring and dose–response modeling of shift work demands is needed to precisely guide clinical practice. Moreover, further research is encouraged to explore shift schedule designs tailored to specific circadian rhythm traits and to evaluate their effects on nurses’ safety behavior as well as their ripple effects on patient outcomes.

## Author Contributions

Z.H. conceptualized and supervised the study, obtained ethics committee approval, and revised and finalized the manuscript. H.Z. contributed to study design, data collection, data processing and analysis, and prepared the initial draft. Y.H. participated in study design, data processing and analysis, and contributed to manuscript editing. Q.L., S.W., and L.L. assisted with participant recruitment and data collection, and contributed to manuscript revision.

## Funding

The authors acknowledge financial support from the Guangxi Natural Science Foundation (2025GXNSFBA069199), the Guangxi Graduate Education Innovation Project (YCBZ2024144), and the Chinese Nursing Association Research Project (ZHKYQ202417).

## Conflicts of Interest

The authors declare no conflicts of interest.

## Supporting Information

Supporting information include additional results supporting the findings of this study: Table S1: Descriptive statistics of continuous study variables. Table S2: Standardized path coefficients for all paths in the three structural equation models (SEMs). Appendix Dataset 1: The dataset used in this study.

## Supporting information


**Supporting Information** Additional supporting information can be found online in the Supporting Information section.

## Data Availability

The dataset used in this study is available in the Supporting Information as Appendix Dataset 1.
